# Addison’s Disease

**Published:** 1883-03

**Authors:** 


Addison's Disease.—Semmola, by new studies, confirms the opinion that Addison's disease must be considered an affection of the central nervous ganglia, and that the anatomical alterations of the suprarenal capsules are not the point of departure of the disease; but where they exist they represent trophic disorders produced by the nervous filaments which preside over the nutrition of these organs, and this he demonstrated at the International Congress in London with drawings which showed the microscopical alterations of some points of the central ganglia and of the dorsal section of the spinal cord, the alterations being a myxomatous transformation of the stroma of the cœliac axis, and leucocytic infiltration of the spinal medulla around the central canal.

He concludes that Addison's disease is a profound disorder of the renal nutrition, determined by the successive alteration of the functions of the sympathetic and of the different nervous centres of organic life (cœliac axis, etc.). And with the help of the physiopathology of the central nervous ganglia, he explains all the successive symptoms of the malady, such as the disorders of digestion, the cachexia, the lowering of the temperature, etc. The discoloration of the skin, when no lesion of the suprarenal capsules can be found, may be attributed to the influence of the sympathetic and central nervous ganglia, which are certainly concerned in the formation of pigment. Not a few cases of melanodermy have occurred a few days after violent moral emotion.

Semmola confirms his assertion by quoting a case of Addison's disease, accompanied by syncope, in which the electrical current, passed from the neck to the epigastrium, did good. In other cases, with the same treatment, there were restoration of strength, increase of the temperature to the normal, improvement in digestion, and, above all, gradual disappearance of pigmentation.—*Lond. Med. Record.*

